# High-density 3-dimensional mapping of an intermittent fasciculoventricular pathway: Intracase comparison of ventricular activations

**DOI:** 10.1016/j.hrcr.2026.03.025

**Published:** 2026-04-02

**Authors:** Komei Mizokami, Kazuo Sakamoto, Tomomi Nagayama, Susumu Takase

**Affiliations:** Department of Cardiovascular Medicine, Kyushu University Hospital, Fukuoka, Japan

**Keywords:** Fasciculoventricular pathway, Intermittent fasciculoventricular pathway, High-density 3-dimensional mapping, Accessory pathway, Preexcitation syndrome, Fixed HV interval

**Key Teaching Points**

• The advent of high-density 3-dimensional (3D) mapping systems could reveal anatomical details of fasciculoventricular pathways (FVPs), a rare preexcitation variant.
• The intermittent FVP enabled precise localization of the ventricular insertion and the block point by comparing high-density 3D maps in the presence and absence of FVP conduction.
• In the presence of FVP conduction, an FVP potential was recorded at the earliest right ventricular (RV) activation site near the His bundle; when FVP conduction was absent, the earliest RV activation site shifted to the apex with proximal FVP block.

## Introduction

Fasciculoventricular pathways (FVPs) are rare variants of preexcitation syndrome in which an accessory pathway connects the His bundle to the ventricular myocardium. Recent advances in 3-dimensional (3D) mapping systems can reveal detailed anatomical findings, and Barrows et al^1^ first visualized the ventricular insertion sites of FVPs using high-density 3D mapping. Here, we report the first case of an intermittent FVP in which 2 distinct ventricular activation maps in the presence and absence of FVP conduction were created via high-density 3D mapping.

## Case report

**Figure 1:**
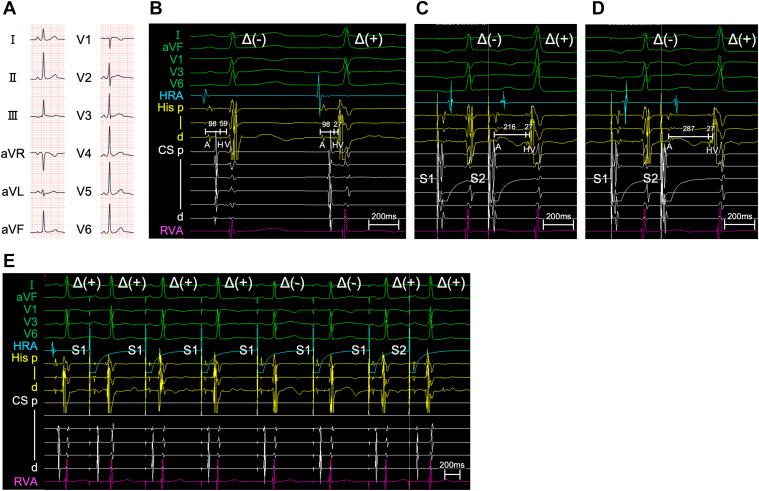
**A:** 12-lead electrocardiogram during manifest delta wave beats. **B:** Intracardiac electrograms in both Δ(−) and Δ(+) beats. **C and D:** During atrial single extrastimulus pacing, the atrial-His interval showed decremental conduction. In contrast, the His-ventricular interval remained at 27 ms both before and after the atrioventricular nodal jump. **E:** During programmed stimulation, Δ(−) and Δ(+) beats appeared randomly. Δ(+) = delta wave–present; Δ(−) = delta wave–absent; CS = coronary sinus; d = distal; His = His bundle; HRA = high right atrium; p = proximal; RVA = right ventricular apex.

A 25-year-old woman was referred for evaluation of ventricular preexcitation. A Wolff-Parkinson-White pattern had been noted on school screening at the age of 13. She had experienced brief (about 1-minute) palpitations, but had never sought care. After childbirth, she was advised to undergo further examination and presented to our hospital. She had no medical history, family history of heart disease, or medications. Echocardiography revealed no evidence of structural heart disease. A 12-lead electrocardiogram obtained in sinus rhythm showed delta-wave polarity of QS in lead V_1_, positivity in lead I/II/aVF, and R≫S in lead Ⅲ, suggesting a right anteroseptal accessory pathway (Figure 1A). The PR interval was 120 ms, which is relatively long for Wolff-Parkinson-White syndrome^2^; the QRS duration was 102 ms.

Electrophysiology study (EPS) was performed to investigate the cause of the patient’s palpitations. Electrode catheters were positioned at the His bundle, right ventricle (RV), and high right atrium–coronary sinus. At the beginning of the EPS, the delta wave was absent on some beats and present on others, consistent with intermittent preexcitation. The atrial-His (AH) interval was 98 ms in both delta wave–absent [Δ(−)] and delta wave–present [Δ(+)] beats, within normal limits. The His-ventricular (HV) interval was 59 ms within normal limits during Δ(−) beats and shortened to 27 ms during Δ(+) beats (Figure 1B). During atrial single extrastimulus pacing, the AH interval showed decremental properties and the HV interval was constant at 27 ms. The delta wave, which reflects the degree of preexcitation, remained unchanged (Figure 1C). After the atrioventricular (AV) nodal jump at S1/S2 750/340 ms, the HV interval remained 27 ms in subsequent atrial single extrastimulus pacing as well (Figure 1D). The AV block was always accompanied by a block at the AH level. During the EPS, Δ(−) and Δ(+) beats randomly appeared without electrophysiological regularity (Figure 1E). Isoproterenol induced delta wave manifestation on every beat during sinus rhythm and pacing maneuvers. Para-Hisian pacing study could not be evaluated because of unstable ventriculoatrial conduction and the failure to achieve pure His-bundle capture, as the paced QRS morphology remained wide and unchanged across a wide range of pacing outputs. Althoughe ventriculoatrial conduction was present, the earliest atrial activation was recorded at the His electrode by V-scan pacing. Programmed atrial and ventricular stimulation did not induce tachycardia. Based on these findings, the diagnosis was dual AV nodal pathways with a bystander intermittent FVP.

**Figure 2:**
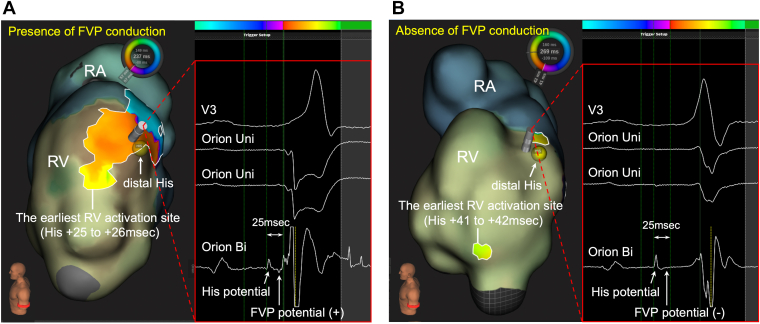
High-density 3-dimensional mapping of the RV and RA using the RHYTHMIA HDx system with an Orion mapping catheter during sinus rhythm: (**A**) in the presence of FVP conduction and (**B**) in the absence of FVP conduction. Bi = bipolar; FVP = fasciculoventricular pathway; RA = right atrium; RV = right ventricular; Uni = unipolar.

High-density 3D mapping was performed using the RHYTHMIA HDx system (Boston Scientific, Marlborough, MA) with an Orion mapping catheter (Boston Scientific, Marlborough, MA). Because FVP conduction was intermittent, we generated 2 high-density 3D maps of the RV and right atrium during sinus rhythm: one in the presence and the other in the absence of FVP conduction (Supplemental Video 1). In Figure 2A showing FVP conduction, the earliest activation site in the RV was located near the His bundle, highlighted by the Lumipoint. This site activated 25–26 ms after the His potential recorded on the distal His electrode (yellow tag). Furthermore, an FVP potential preceding the ventricular electrogram was recorded at the annotation tag. In contrast, in the absence of FVP conduction, the earliest RV activation site highlighted by the Lumipoint was located at the apex, with a delay of 41–42 ms from the His potential (Figure 2B). In addition, no FVP potential was recorded at the annotation tag where the FVP was presumed to be, indicating that conduction through the FVP was blocked.

Ablation targeting the ventricular insertion site of the FVP was considered because administration of isoproterenol unmasked robust anterograde conduction over the FVP, raising a theoretical risk of wide QRS tachycardia (eg, preexcited atrial arrhythmias). However, because no clinical tachycardia was documented or induced and given the potential risk of damaging the AV conduction system, we ultimately deferred ablation. The arrhythmia responsible for the patient’s palpitations could not be identified, and we elected outpatient follow-up. No recurrence of palpitations was observed during 1.5 years of follow-up.

## Discussion

To our knowledge, this is the first case of an intermittent FVP in which 2 distinct ventricular activation maps in the presence and absence of FVP conduction were created using high-density 3D mapping. Although there have been reports on electrophysiological characteristics and anatomical localization of FVPs using fluoroscopy or low-density 3D mapping, the advent of high-density 3D mapping systems has made more precise evaluation possible. In 2025, detailed visualization of the ventricular insertion of an FVP was first reported using high-density 3D mapping systems, which clarified the connection of the FVP with millimeter precision.^1^ Although the previous report visualized maps with constant FVP conduction, we successfully created 2 distinct maps from the same patient both in the presence and absence of FVP conduction. This rare opportunity may offer further insights into FVP pathophysiology.

This case was diagnosed as an FVP because it fulfilled all of the following criteria: (1) a normal baseline AH interval, (2) a short and fixed baseline HV interval (<35 ms), and (3) AH interval prolongation without changes in the HV interval or in the degree of preexcitation during atrial extrastimulus testing.^2^ Notably, the HV interval remained unchanged even after an AV nodal jump was observed under atrial extrastimulus pacing, which was consistent with the characteristics of an FVP connecting the His bundle to the ventricle. However, pure His-bundle capture could not be achieved during para-Hisian pacing and we were unable to demonstrate the characteristic findings expected in an FVP: a paced QRS morphology during pure His-bundle capture that resembles the QRS morphology during sinus rhythm.^3^

Various conduction characteristics of FVPs, including intermittent conduction properties, have been reported.^3^ At the beginning of the EPS, Δ(−) and Δ(+) beats coexisted randomly during sinus rhythm. During atrial extrastimulus testing from various pacing sites, delta waves of S1 randomly appeared without any regularities whereas S2 always had delta waves. The random appearance of a delta wave with S1 was considered to indicate weak conduction ability of the FVP. In contrast, a delta wave was consistently present with S2 even when the His-His interval in S1S2 was shorter than that in the basic cycle length (S1S1). This electrophysiological phenomenon suggested the possibility of supernormal conduction through the FVP.^4^ Administration of isoproterenol induced manifest delta waves on every beat during sinus rhythm as well as atrial extrastimuli, indicating that the conduction ability of the FVP was enhanced by β-adrenergic stimulation.^3^

We next consider the anatomical location of the FVP insertion. In the presence of FVP conduction, the earliest activation site in the RV was near the His bundle at the 1 o’clock position of the tricuspid annulus, consistent with the accessory pathway site predicted from the 12-lead electrocardiogram. The earliest RV activation occurred 25 ms after the His potential. This timing closely matched the HV interval measured during the EPS, confirming this as the true site of earliest ventricular activation. Furthermore, an FVP potential was recorded immediately preceding RV potential in the earliest RV activation site. In the absence of FVP conduction, the earliest activation site was located at the RV apex, reflecting normal conduction through the right bundle, and the onset of RV activation was later than that with FVP conduction. Moreover, when conduction was absent, no FVP potential was observed at the site where it was expected. This finding suggests that the FVP was blocked at a proximal site near the His bundle. In contrast, left ventricular (LV) connections of FVPs have been reported in about 12% of cases.^2^ Although we could not determine the precise course and complete 3D pathway including the LV because we did not map the LV, based on the findings described above, the insertion site of the FVP in our case could potentially be located in the RV.

Finally, we discuss ablation of the FVP. To our knowledge, we are not aware of any published reports describing ablation specifically targeting an FVP, likely for 2 reasons. First, FVPs typically do not participate in reentrant tachycardia. Second, ablation in the para-Hisian region carries a substantial risk of damaging the AV conduction system.^5^ In our case, although the FVP could not be entirely ruled out as a potential contributor to the patient’s palpitations, the decision was made to forgo ablation in view of the favorable prognosis of FVPs and the significant risk of AV block.

## Conclusion

The intermittent nature of FVP conduction provided a unique opportunity to identify the precise insertion site and block point by comparing high-density 3D maps created in the presence and absence of FVP conduction.

## Disclosures

The authors have no conflicts of interest to disclose.
