# Supplementary material for: High-density 3-dimensional mapping of an intermittent fasciculoventricular pathway: Intracase comparison of ventricular activations
Source: HeartRhythm Case Rep. 2026 Apr 2;12(6):685–8. doi: 10.1016/j.hrcr.2026.03.025 (PMC13270938; doi:10.1016/j.hrcr.2026.03.025)
Supplement: Supplementary Legends [file mmc2.docx]

**Supplementary Video and Legends**


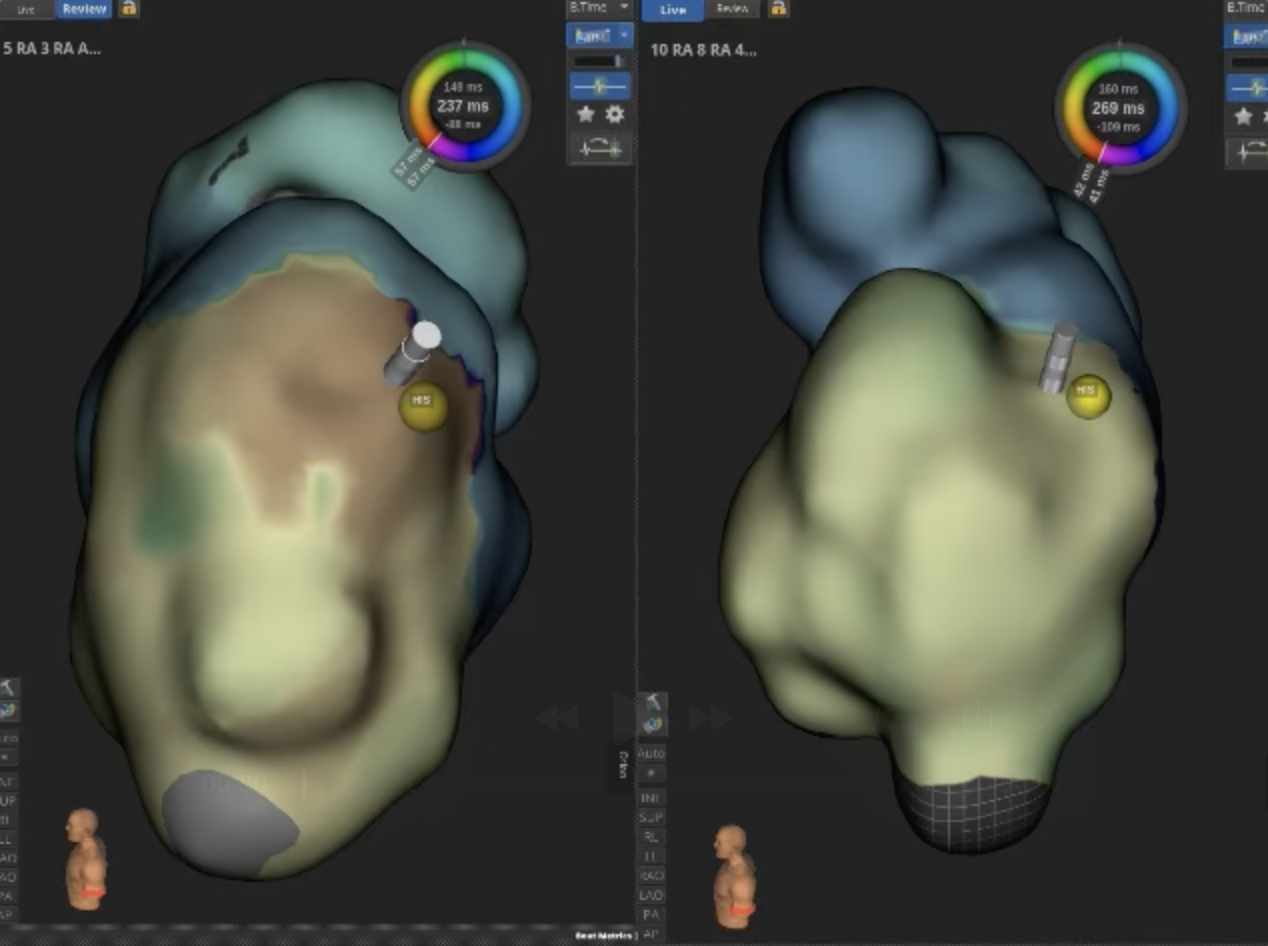


The video displays two distinct high-density 3D maps of right atrium and right ventricular activation: the left panel in the presence and the right panel in the absence of fasciculoventricular pathway (FVP) conduction.
